# Neurosurgery training camp for medical student: experience of the Turkish neurosugery academy and Bursa Uludag University

**DOI:** 10.3389/fsurg.2024.1433780

**Published:** 2024-08-02

**Authors:** H. Setenay Unal, Mevlut Okan Aydin, Esma Bilgic, Pınar Eser, Zuleyha Alper, M. Ozgur Taskapılıoğlu, M. İlker Kafa, Hasan Kocaeli, Seref Dogan, Selcuk Yılmazlar, Ahmet Bekar, Zeki Sekerci, Kaya Aksoy

**Affiliations:** ^1^ Department of Neurosurgery, Bursa Uludag University School of Medicine, Bursa, Turkiye; ^2^ Department of Medical Education, Bursa Uludag University School of Medicine, Bursa, Turkiye; ^3^ Bursa Uludag University School of Medicine, Bursa, Turkiye; ^4^ Department of Anatomy, Bursa Uludag University School of Medicine, Bursa, Turkiye; ^5^ Department of Neurosurgery, President Turkish Neurosurgery Academy, Medipol University School of Medicine, Istanbul, Turkiye; ^6^ Private Practitioner, Head of Turkish Neurosurgery Academy, Bursa, Turkiye

**Keywords:** neurosurgery, training camp, boot camp, student education, surgical training

## Abstract

**Introduction:**

To highlight the importance of hands-on experiences and mentorship in shaping the future workforce of specialized medical professionals via a Neurosurgery Training Camp.

**Methods:**

Responses of the questionnaire regarding the Neurosurgery Training Camp organized by Bursa Uludag University's Faculty of Medicine and the Turkish Neurosurgery Academy were reviewed retrospectively. A one-day program was organized to introduce neurosurgery to medical students. During the camp, the students participated in interactive presentations delivered by faculty members, had lunch together, became acquainted with neurosurgical tools and technologies, and performed interventions. With pre and postworkshop questionnaire, student's expectations and thoughts about camp was evaluated.

**Results:**

Forty-one students from 10 medical schools, spanning every year of study, attended the camp. Approximately 39% of the attendees (*n* = 16) were women and 61% (*n* = 25) were men. The post-workshop survey results demonstrated that 73% of the students (*n* = 30) were inclined to pursue a career in neurosurgery after the camp, 21.9% (*n* = 9) remained undecided, and 4.8% (*n* = 2) chose not to pursue neurosurgery. Feedback from the post-workshop questionnaire highlighted that all students perceived the camp as beneficial in providing insights into their future careers and aiding in making a decision regarding their career paths.

**Discussion:**

The neurosurgical training camp effectively inspired and educated medical students about the discipline of neurosurgery. Furthermore, the camp effectively altered the career aspirations and perceptions of neurosurgery among the participating students.

## Introduction

The neurosurgery specialty is a lifelong and arduous process that encompasses various challenges. In Turkey, after the completion of a six-year medical school program, physicians face a challenging exam called the “Medical Specialty Exam.” Based on their performance in this assessment, they are granted the opportunity to choose their respective specializations. Nevertheless, this examination must not be regarded as the sole measure of a physician's level of enthusiasm towards the realm of neurosurgery. During the internship period of their six-year medical curriculum, individuals are engaged in the neurosurgery department for approximately one week.

Recently, there has been a global trend of doctors displaying a reluctance towards pursuing surgical specialties (1). This hesitancy can be attributed to several factors such as the extended duration of residency programs, demanding night shifts, instances of workplace harassment, and other issues within the medical education system (2–4). Consequently, the number of students migrating abroad after completing their medical education has increased, and the inclination towards pursuing specialty training within Turkey has declined (5, 6).

Training programs were established to enhance the surgical skills of medical students (7, 8). However, training camps, particularly in the field of neurosurgery, aimed at catering to the requirements of medical students have recently emerged. These initiatives effectively guide students in making informed decisions regarding their specialization choices and provide them with a comprehensive understanding of the intricate process (9–11).

The Turkish Neurosurgery Academy oversaw the organization of the Neurosurgery Training Camp by the Faculty of Medicine at Bursa Uludag University, in collaboration with the Departments of Neurosurgery and Medical Education. This event was specifically designed for medical students and graduate practitioners, with the primary objectives of assessing their knowledge and skills in the field of neurosurgery, aiding them in making informed choices regarding their specializations, facilitating their engagement in simple surgical interventions, and offering them a glimpse into the potential prospects within this department.

Recognizing the aspirations of students who intend to pursue opportunities abroad, pre-graduation training camps within the field of neurosurgery were established. The fundamental reasoning behind this initiative was to motivate these students to remain in Turkey, instill a feeling of optimism regarding their prospects, and expand their horizons in the process.

In this study, we aimed to highlight the importance of hands-on experiences and mentorship in shaping the future workforce of specialized medical professionals via a Neurosurgery Training Camp.

## Materials and methods

The Turkish Neurosurgery Academy, a division of the Turkish Neurosurgical Society, held the second Neurosurgery Training Camp in 2023. The camp took place at the Bursa Uludag University's Good Medical Practices and Simulation Center, and it was attended by 22 faculty members and 41 students from various towns and universities. Before the camp began, all students had to provide motivation letters outlining their interest in neurosurgery, their previous experience in the subject during medical school, and their expectations for the camp.

Students were assessed using open-ended questions about their interest in neurosurgery before and after the camp (Table 1). The students' answers to open-ended questions and the positive and negative changes in their decisions about choosing Neurosurgery as a career were statistically evaluated.

**Table 1:** Pre and postworkshop questions.

Questions	Preworkshop			Postworkshop		
	Yes (%)	No (%)	Undecided (%)	Yes (%)	No (%)	Undecided (%)
1. Are you interested in the neurosurgery?	87,8 (*n*:36)	7,3 (*n*:3)	4,87 (*n*:2)	95,1 (*n*:39)	4,87 (*n*:2)	0
2. Do you think neurosurgery is a suitable department for you?	87,8 (*n*:36)	7,3 (*n*:3)	4,87 (*n*:2)	95,1 (*n*:39)	4,87 (*n*:2)	0
3. Do you think this camp will have an impact on your choice of Neurosurgery department?	95,1 (*n*:39)	4,87 (*n*:2)	0			
4. Is this camp helpful for your choice?				95,1 (*n*:39)	4,87 (*n*:2)	0

The morning sessions included oral presentations, lectures, and interactive discussion panels. Twenty-two professors discussed their experiences on different subjects with the students (Table 2). At noon, the faculties and students ate together. Each table consisted of 1 professors, a neurosurgery resident from Bursa Uludag University School of Medicine, Department of Neurosurgery, and 6–7 randomly chosen students. At lunch, each student interacted with other students and teachers, exchanging questions and experiences.

**Table 2:** Summary of the lectures given.

1. Is neurosurgery a passion? 2. Why science and neurosurgery should be together 3. What does a professional organization bring to neurosurgery? 4. Neurosurgery subspecialties 5. National and international neurosurgery training 6. Contribution of other branches of neurological sciences to neurosurgery: Neuroradiology 7. Neuropathology: Contribution of genetics and molecular biology to tumor surgery 8. Neuroanatomy: New learning models 9. Pain, epilepsy and stereotactic 10. Contemporary technical advances in neurosurgery: The changing face of neurosurgery 11. Basic principles of peripheral nerve surgery 12. Head trauma 13. Minimally invasive cranial surgery 14. Minimally invasive spinal surgery 15. “BOARD” accreditation in neurosurgery 16. International “fellowship” programs

**Figure 1:**
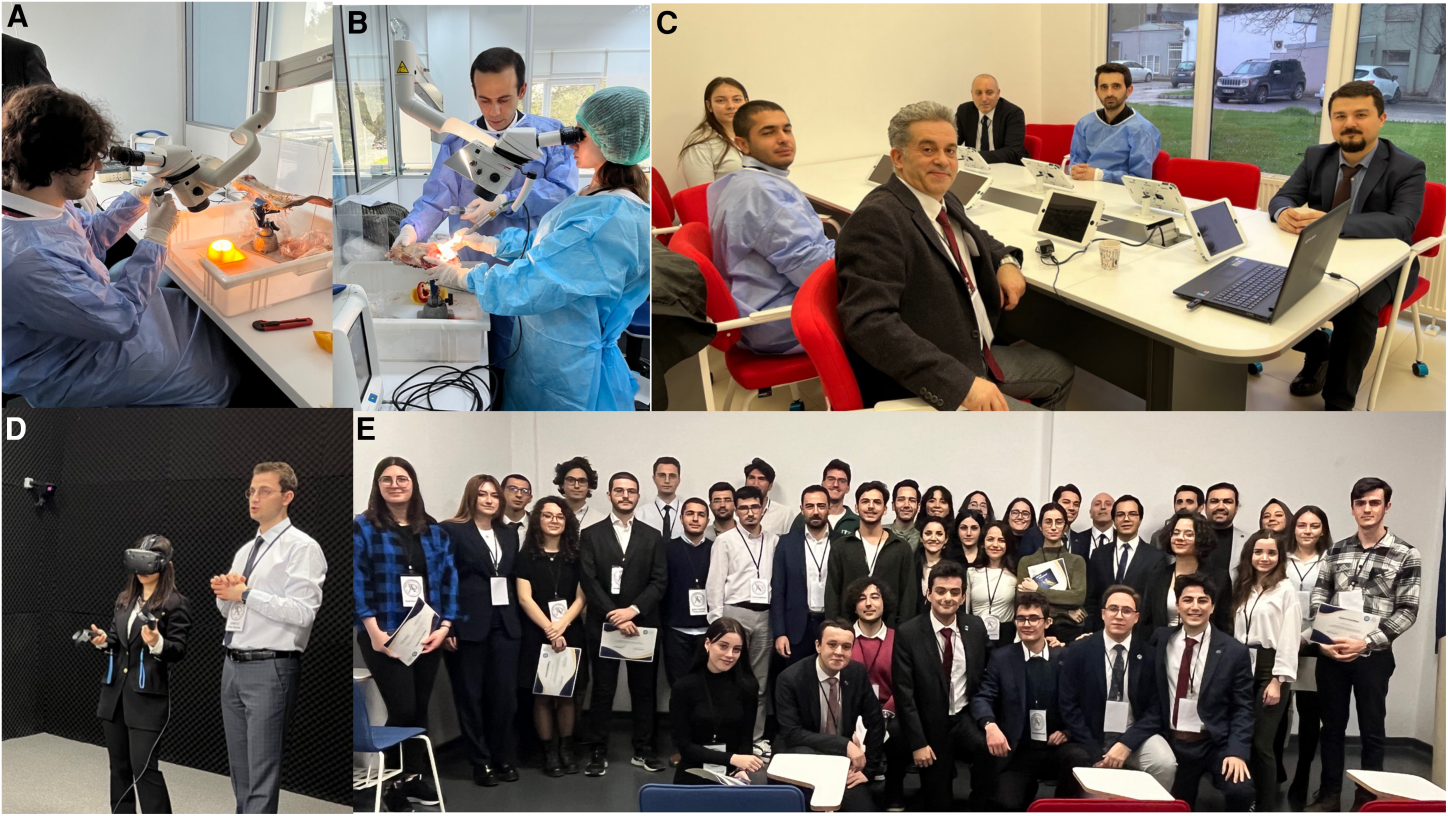
Different sections of the camp. (**A, B**) Section where the microscope and electrical bone drill were used. (**C**) E-learning section. (**D**) 3D anatomy section. (**E**) Students with their program certificate.

During the afternoon sessions, students were practically guided by faculty members on utilizing microscopes in neurosurgery, recognizing surgical instruments and devices, and determining locations through stereotactic procedures. This session was subdivided into three segments which were held in different classrooms. During Part 1 of the session, the students performed spinal surgeries such as laminotomy and laminectomy under the guidance of three distinct professors at animal cadaveric spine (sheep). Part 2 of the session involved students receiving hands-on training in surgical knots, dural sutures, microscope use, burr-hole drilling, craniotomy, and scalp-meninges-dura opening at animal cadaveric scalp (sheep). They were supervised by three different instructors. Part 3 of the session covered surgical positions in neurosurgery, characteristics of the surgical table, and utilization of neuronavigation, endoscopes, cavitational ultrasonic surgey aspitators (CUSA), aspirators, and bipolar/unipolar devices (Table 3) (Figure 1A–E).

**Table 3:** Devices/tools used by the students when performing procedures.

• 3D anatomy • E-learning • Electrical bone drill system (Midas, …) • Microscope • Mono-bipolar • Cavitron ultrasonic surgical aspirator (CUSA) • Navigation • Mayfield skull clap

All the students were awarded a certificate of participation by the members of the Department of Neurosurgery, Uludag University School of Medicine.

## Results

Forty-one students from 10 medical schools participated in our camp. Having participants from 10 different medical schools ensured that there was a diverse range of ideas and experiences, which enhanced the learning atmosphere. The participant gender distribution was balanced (39% women and 61% men) and highlighted the inclusivity of neurosurgical education. At our camp, 46% (*n* = 19) of the participants were fourth-year students, 19.5% (*n* = 8) were fifth-year students, and 12.1% (*n* = 5) were sixth-year students. Approximately 78% of the students were in the final 3 years of their studies (Table 4). The workshop's appeal to a wide range of learners at different stages of their medical education is highlighted by the distribution of students throughout multiple academic years, from first-year to sixth-year students.

**Table 4:** Demographic of the attendees.

Characteristic	Number
Sex	
Male	16
Female	25
Year in medical school	
1	2
2	5
3	2
4	19
5	8
6	5
Medical school name/city	
Uludag University/Bursa	17
Bahcesehir University/Istanbul	11
Cerrahpasa University/ Istanbul	1
Hacettepe University/Ankara	1
Atatürk University/Erzurum	4
Erciyes University/Kayseri	1
Okan University/ Istanbul	3
Kocaeli University/Kocaeli	1
Ufuk University/ Istanbul	1
Health and Technology University/ Istanbul	1

Post-workshop survey results indicated that 73% (*n* = 30) of the students chose to pursue a career in neurosurgery following the camp, 21.9% (*n* = 9) were undecided, and 4.8% (*n* = 2) decided against pursuing neurosurgery. Two neurosurgery aspirants changed their minds and opted not to pursue the career path after completing the camp. Thus, the camp was beneficial in helping them make a decision.

The postworkshop questionnaire revealed that all students found the camp beneficial in helping them understand what to expect in neurosurgery and making decisions about their career paths.

The answers to the open-ended question highlighted two prominent themes. The first refers to the transmission of knowledge from an expert, while the second pertains to the practical knowledge gained in the specific field of expertise.

One participant expressed that the transfer of experts' experience to career choice had a positive impact. They stated, “Observing the dedication and way of life of faculty members at this level made me reassess everything.” A different attendee highlighted the significance of sharing expertise through presentations and organized discussions at the camp, stating, “Having the opportunity to engage in one-on-one conversations with faculty members during meals and practices and receiving their perspectives was extremely valuable.”

One participant expressed that the transfer of experts' experience to career choice had a positive impact. They stated, “Observing the dedication and way of life of faculty members who have achieved this level made me reassess everything.” Another attendee highlighted the significance of sharing knowledge through presentations and organized discussions at the camp, stating, “Having the opportunity to engage in one-on-one conversations with faculty members during meals and practices and hear their perspectives was extremely valuable.”

The majority of participants concurred that their exposure to medical practices during their specialization period had a positive impact on their career decision. Regarding this matter, a participant expressed, “The practical component of the program proved to be highly beneficial, as it provided us with valuable educational experiences that we may not have encountered prior to completing our studies.” Another participant highlighted the significance of engaging in practical activities during career camps, stating, “I had the chance to explore various technologies, which greatly enhanced my understanding of brain and neurosurgery.”

## Discussion

Recently, there has been an increase in unwillingness among medical professionals globally to choose surgical specialties. Factors like extended residency years, rigorous night shifts, workplace harassment, and systemic problems in medical education institutions have led to this pattern (8, 11). Thus, more medical students are looking to migrate abroad after completing their medical education, and their interest in pursuing specialist training in their home countries, such as Turkey, is decreasing.

Events featuring experts and faculty who share their experiences have a significant impact on career choices in medicine. These events provide valuable insights, mentoring, and networking opportunities. Faculty members' reflective narratives can stimulate trainees to engage in introspection and social interaction, motivating them to emulate exemplary figures and mold their professional identities (12). Virtual events, such as webinars and roundtable sessions, are highly effective in enhancing students' exposure to a wide range of specialties, enhancing their understanding of educational pathways, and influencing their decision-making processes regarding their careers demonstrated (13, 14). These events are particularly vital during the COVID-19 pandemic, when traditional face-to-face interactions and clinical exposures are restricted (15). By prioritizing the humanistic aspects of medicine, inclusion has the potential to enhance students' comprehension, empathy, and ultimately impact their career decisions (16, 17).

To address these difficulties and promote the development and retention of skilled individuals in the neurosurgical field, Bursa Uludag University's Faculty of Medicine partnered with the Turkish Neurosurgery Academy to host a Neurosurgery Training Camp. This program was designed to offer medical students and practicing graduates a direct chance to delve into the complexities of neurosurgery, assess their suitability for the specialty, and acquire knowledge regarding the residency process and potential career opportunities in the area.

The camp provided participants with a practical experience, along with mentorship from experienced professionals, to enhance their awareness of the difficulties and benefits of a career in neurosurgery. The communal lunchtime atmosphere allowed professors, neurosurgery residents, and students to network, mentor, and share ideas and experiences. The afternoon sessions involved rotating among seven distinct classes that helped students gain practical experience with various neurosurgical instruments and techniques while being supervised by experts.

The Neurosurgery Training Camp and similar efforts are crucial for integrating academic knowledge with practical implementation. Participants can perform basic surgical procedures under supervision, allowing them to develop confidence and skill.

The postworkshop survey findings demonstrate the camp's significant influence on students' career goals. Approximately 73% of the participants exhibited an interest in pursuing a career in neurosurgery after the camp. This high percentage indicates the workshop's success in inspiring and pushing students to pursue neurosurgery, potentially helping to alleviate workforce shortages and generate interest in a specialized medical field.

Initiatives such as the Neurosurgery Training Camp help medical students and practitioners discover their interests and evaluate their suitability for neurosurgery. This contributes to creating a healthcare workforce that is well-informed and driven. By adopting proactive actions, stakeholders can tackle the issues in medical education and specialist training to maintain a sustainable supply of trained practitioners in essential areas such as neurosurgery.

## Study limitation

The short-term thoughts of the students after the camp were learned, but since they had not graduated yet, it could not be evaluated whether the students chose Neurosurgery during the long-term follow-up. Follow-up of students participating in the camp will continue. These camp results will be compared with subsequent groups to obtain comprehensive information. However, only students who wanted to specialize in neurosurgery were included in the camp. This can be considered as an issue that may affect the objectivity of the research.

## Conclusion

Students in medical school were effectively inspired and educated about the discipline of neurosurgery via the Neurosurgery Training Camp. The students' career goals and their opinions of neurosurgery were successfully changed by the camp. This was accomplished via a combination of interactive presentations, hands-on experiences, and opportunity to network with other individuals. To adequately foster and support future neurosurgeons, it is vital to continue evaluating and refining educational efforts of this kind. The in-depth analysis of the various components and outcomes of the camp allows us to conclude that the Neurosurgery Training Camp was highly effective in achieving its goals and shaped the students' perceptions on neurosurgery as a career path.

## Data Availability

The raw data supporting the conclusions of this article will be made available by the authors, without undue reservation.

## References

[1] Hoffmann H Dell-Kuster S Rosenthal R. Medical students’ career expectations and interest in opting for a surgical career. Swiss Med Wkly. (2014) 144:w13932. 10.4414/smw.2014.13932 24567266

[2] Boyle E Healy D Hill AD O'Connell PR Kerin M McHugh S Career choices of today’s medical students: where does surgery rank? Ir J Med Sci. (2013) 182(3):337–43. 10.1007/s11845-012-0882-x 23242574

[3] Peel JK Schlachta CM Alkhamesi NA. A systematic review of the factors affecting choice of surgery as a career. Can J Surg. (2018) 61(1):58–67. 10.1503/cjs.008217 29368678 PMC5785290

[4] McLennan S Purich K Verhoeff K Mador B. Attitudes of Canadian medical students towards surgical training and perceived barriers to surgical careers: a multicentre survey. Can Med Educ J. (2023) 14(5):71–6. 10.36834/cmej.74694 38045085 PMC10690001

[5] Kaya AE Aktürk BE Aslan E. Factors predicting the motivation to study abroad in Turkish medical students: a causal investigation into the problem of brain drain. J Health Sci Med. (2023) 6:526–31. 10.32322/jhsm.1253308

[6] Sancak B Selek SN Sarı E. Depression, anxiety, stress levels and five-factor personality traits as predictors of clinical medical students’ migration intention: a cross-sectional study of brain drain. Int J Health Plann Manage. (2023) 38(4):1015–31. 10.1002/hpm.3646 37062888

[7] Klingensmith ME Brunt LM. Focused surgical skills training for senior medical students and interns. Surg Clin North Am. (2010) 90(3):505–18. 10.1016/j.suc.2010.02.004 20497823

[8] O'Herrin JK Lewis BJ Rikkers LF Chen H. Medical student operative experience correlates with a match to a categorical surgical program. Am J Surg. (2003) 186(2):125–8. 10.1016/s0002-9610(03)00188-0 12885602

[9] Radwanski RE Winston G Younus I ElJalby M Yuan M Oh Y Neurosurgery training camp for sub-internship preparation: lessons from the inaugural course. World Neurosurg. (2019) 127:e707–16. 10.1016/j.wneu.2019.03.246 30947014

[10] Yearley AG Ng PR Gupta S Cosgrove GR Mooney MA. Utility of a pilot neurosurgical operative skills boot camp in medical student training. World Neurosurg. (2022) 166:e551–60. 10.1016/j.wneu.2022.07.065 35870784

[11] Ashraf M Ismahel H Lub S Gardee A Evans VE Middleton EES Role of a medical student neuro-society organized neurosurgical conference: the Glasgow neuro experience. Surg Neurol Int. (2023) 24(14):70. 10.25259/SNI_755_2022 PMC9990638 36895225

[12] Khoo SM Wong XLS. When faculty tell tales: how faculty members’ reflective narratives impact residents’ professional identity formation. Acad Med. (2022) 97(3):385–8. 10.1097/ACM.0000000000004256 34323858

[13] Nguyen AX Pur DR Lo C Gottlieb C Hardy I. Experiences from a national webinar with recently matched Canadian ophthalmology residents for medical students. Can J Ophthalmol. (2022) 57(4):e131–3. 10.1016/j.jcjo.2021.10.004 34785149

[14] Hogan D Geary S Hennessey DB. The impact of a novel surgical forum, ‘virtual surgical speed dating’, on career perception for medical students: a pilot study. Ir J Med Sci. (2023) 192(6):2987–92. 10.1007/s11845-023-03361-2 37055703 PMC10101731

[15] Shakir T Matwala K Lovett B. SP7.2 Student reported outcomes of a novel virtual medical work experience: results from an international cohort. Br J Surg. (2022) 109:25. 10.1093/bjs/znac247.075

[16] Pirjani R Rabiei M Alipour S Hosseini R Shahvari Z. Promoting professionalism in practice: using patients’ lived experiences. Med Educ. (2019) 53(5):505. 10.1111/medu.13863 30891829

[17] Player E Gure-Klinke H North S Hanson S Lane D Culyer G Humanising medicine: teaching on tri-morbidity using expert patient narratives in medical education. Educ Prim Care. (2019) 30(6):368–74. 10.1080/14739879.2019.1670097 31580229

